# Supplementary material for: The essential kinase TgGSK regulates centrosome division and endodyogeny in Toxoplasma gondii
Source: bioRxiv. 2024 Sep 27:2024.09.27.615374. Preprint. [Version 1] doi: 10.1101/2024.09.27.615374 (PMC11463552; doi:10.1101/2024.09.27.615374)
Supplement: 1 [file NIHPP2024.09.27.615374V1-supplement-1.pdf]

bioRxiv preprint doi: https://doi.org/10.1101/2024.09.27.615374; this version posted September 27, 2024. The copyright holder for this preprint (which was not certified by peer review) is the author/funder, who has granted bioRxiv a license to display the preprint in perpetuity. It is made available under aCC-BY 4.0 International license.

Purpose	Name	Sequence
Generate pSag1-Cas9-U6-sgGSK-TG-HXG CRSPR/Cas9 plasmid for endogeneous tagging	GSK-tg-sgRNA.For	GTCTTTTTTTGTTTTAGAGCTAGAAATAGC
	GSK-tg-sgRNA.Rev	CGACAGCTGCAACTTGACATCCCCATTTAC
Amplify 3xHA-DHFR cassette from the plasmid pLIC-3xHA-DHFR	GSK-TG-insert.For	ATGTATTCCGAAGCATATCGCCAGTGCAAACAACC GTGGCTTAATTAAAATTGGAAGTGGAGG
	GSK-TG-insert.Rev	AGCATAAGAGAAGCTCCCCATCCCTAGTAGGTGTA GGGAGGTTTTCCCAGTCACGACG
Generate pSag1-Cas9-U6-sgGSK-TATI-HXG CRSPR/Cas9 plasmid for endogeneous tagging	GSK-TATI-sgRNA.For	AAGAAGGGGTGTTTTAGAGCTAGAAATAGC
	GSK-TATI-sgRNA.Rev	TCCTTCGTCCAACTTGACATCCCCATTTAC
Amplify TATi cassette from the plasmd 5'COR-pT8TATi1-HX-tetO7S1mycNtCOR.dna	GSK-TATI-insert.For	CACTCATCTTTTCCTGGCCTTTGTCGAGAAGGCAG AAGTCTCTTCTCATGTTTGCGGATCCG
	GSK-TATI-insert.Rev	CTACTCTTCTGAGCAGCTGCGGGATCGTACTGCG GGTCCGGCATTTTGATATCCCTAGGAATTCACTC

Supplemental table 1. Primers used in this work. Sequences are 5’ to 3’.

bioRxiv preprint doi: https://doi.org/10.1101/2024.09.27.615374; this version posted September 27, 2024. The copyright holder for this preprint (which was not certified by peer review) is the author/funder, who has granted bioRxiv a license to display the preprint in perpetuity. It is made available under aCC-BY 4.0 International license.

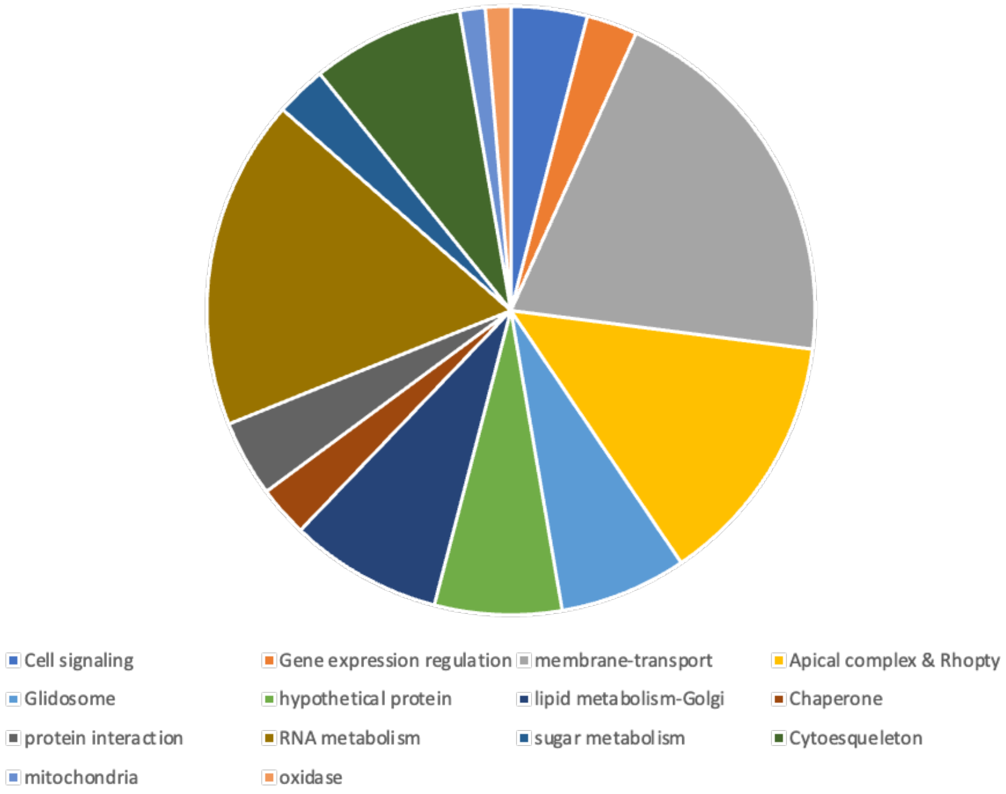

Supplemental figure S1. Classification of hypothetical proteins with TgGSK-dependent phosphopeptides. Hypothetical proteins with peptides differentially phosphorylated in the knockdown vs the parental were analyzed based on annotations in the *Toxoplasma* genome database or homology to proteins in other Apicomplexan species. Additionally, the protein domains were analyzed based on known conserved functions. Some of these proteins remained classified as hypothetical.
